# Adrenomedullin protects rat dorsal root ganglion neurons against doxorubicin-induced toxicity by ameliorating oxidative stress

**DOI:** 10.22038/ijbms.2020.45134.10514

**Published:** 2020-09

**Authors:** Amir Mahmoodazdeh, Sayed Mohammad Shafiee, Mohsen Sisakht, Zahra Khoshdel, Mohammad Ali Takhshid

**Affiliations:** 1 Department of Biochemistry, School of Medicine, Shiraz University of Medical Sciences, Shiraz, Iran; 2 Autophagy Research Center, Shiraz University of Medical Sciences, Shiraz, Iran; 3 Diagnostic Laboratory Sciences and Technology Research Center, School of Paramedical Sciences, Shiraz University of Medical Sciences, Shiraz, Iran

**Keywords:** Adrenomedullin, Dorsal root ganglia, Doxorubicin, Inflammation, Oxidative stress

## Abstract

**Objective(s):**

Despite effective anticancer effects, the use of doxorubicin (DOX) is hindered due to its cardio and neurotoxicity. The neuroprotective effect of adrenomedullin (AM) was shown in several studies. The present study aimed to evaluate the possible protective effects of AM against DOX-induced toxicity in dorsal root ganglia (DRGs) neurons.

**Materials and Methods:**

Rat embryonic DRG neurons were isolated and cultured. The effect of various concentrations of DOX (0.0 to 100 µM) in the absence or presence of AM (3.125 -100 nM) on cell death, apoptosis, oxidative stress, expression of tumor necrosis-α (TNF-α), interleukin1- β (IL-1β), inducible nitric oxide synthase (iNOS), matrix metalloproteinase (MMP) 3 and 13, and SRY-related protein 9 (SOX9) were examined.

**Results:**

Based on MTT assay data, DOX decreased the viability of DRG neurons in a dose and time-dependent manner (IC_50_=6.88 µm) while dose-dependently, AM protected DRG neurons against DOX-induced cell death. Furthermore, results of annexin V apoptosis assay revealed the protective effects of AM (25 nm) against DOX (6.88 µM)-induced apoptosis and necrosis of DRG neurons. Also, AM significantly ameliorated DOX-induced oxidative stress in DRG neurons. Real-time PCR results showed a significant increase in the expression of TNF-α, IL-1β, iNOS, MMP 3, and MMP 13, and a decrease in the expression of SOX9 following treatment with DOX. Treatment with AM (25 nM) significantly reversed the effects of DOX on the above-mentioned genes expression.

**Conclusion:**

Our findings suggest that AM can be considered a novel ameliorating drug against DOX-induced neurotoxicity.

## Introduction

Doxorubicin (DOX, Adriamycin) is amongst the widely used chemotherapeutic drugs (1). Increased generation of reactive oxygen and nitrogen species (ROS and RNS) that leads to DNA and cell membrane damages and disturbance in DNA replication is the underlying anti-cancer effect of DOX (2). The use of DOX is usually associated with the deleterious effects on several organs including the central nervous system (CNS) (3). Significant impairment in the learning abilities and memory loss were reported in patients undergoing DOX-based chemotherapy (2, 4). Destruction of dorsal root ganglion (DRG) cells is another neurotoxic effect of DOX, which was shown both in animal models (5) and human studies (6). 

It was shown that primarily DOX-induced toxicity, but not exclusively, mediated through ROS and RNS generation (7, 8). Accumulation of ROS and NOS triggers a sequence of damaging processes associated with lipid peroxidation, DNA damage, and mitochondrial dysfunction, causing cellular damage, energy deficit, and induction of apoptotic or necrotic cell death (7). Meanwhile, elevated superoxide anion level is able to increase the circulating level of tumor necrosis factor-alpha (TNF-α), which passes through the blood-brain barrier (BBB) by triggering the production of pro-inflammatory cytokines, oxidative and nitrosative damage, and eventually neural death (9). Furthermore, DOX and its metabolites strongly bond to free iron ion or interact with proteins that transport and bind to intracellular iron that deregulates iron homeostasis and induces ROS production (2). Several other mechanisms were shown to be involved in cytotoxicity of DOX, such as dysregulation of lipid signaling pathways (10), induction of key stress kinases (11), activation of inflammatory cytokines, including TNF-α and interleukin1- β (IL-1β) (12, 13) and secondary inflammatory mediators, such as matrix metalloproteinases (MMPs) (14, 15).

Adrenomedullin (AM), a neuropeptide with 52 residues, is widely expressed in various body organs including CNS (16). In the CNS, high expression of AM was reported in DRG and dorsal horn of the spinal cord, where it is involved in the transmission of nociceptive impulses (17, 18). Biological effects of AM are exerted through AM1 and AM2 receptors, combination of calcitonin receptor-like receptor (CLR) and receptor activity modifying proteins (RAMPs) type 2 or 3 (19). Due to structural similarities with calcitonin gene-related peptide (CGRP), a part of AM function is mediated through CGRP receptors (20). Abundant AM binding site and high expression of both components of AM receptors, CLR, and RAMP2 and 3 have been shown in DRG and the spinal cord, both on the pre and postsynaptic membranes, suggesting auto and paracrine actions of AM at the DRG level (10). AM is primarily known for its vasorelaxant and hypotensive effects (20). It is also known as an anti-inflammatory neuropeptide with the ability to decrease neurogenic inflammation by blocking the inflammatory cells’ function (9). Inhibition of IL-1β-induced rheumatoid synovial fibroblast proliferation and decrease in the production of MMPs, cyclooxygenase, and prostaglandin E2 are the possible mechanisms involved in the anti-inflammatory function of AM (21). Furthermore, the protective properties of AM were reported against several pathological conditions including ischemic brain damage (22), Pyroptosis of Leydig cells (23), heart failure (24), pulmonary and renal diseases (25), sepsis (26), and ulcerative colitis in the animal models (27). Antioxidant effect of AM was indicated in several toxic conditions, such as lead toxicity (28), cerulean-induced acute pancreatitis (29), and DOX-induced cardiac damage (30). 

To the best of our knowledge, the protective effect of AM against DOX-induced toxicity in the DRG neurons has not been investigated. Hence, the current study was designed to clarify the possible protective effect of AM against DOX-induced cell death, oxidative stress, and expression of inflammatory mediators in the isolated rat embryonic DRG neurons. Therefore, the effects of AM on the DOX-induced toxicity including cell death, apoptosis, oxidative stress, and expression of inflammatory cytokines, such as TNF-α and IL-1β and MMPs, were examined. 

## Materials and Methods

The experimental protocols were approved by the Institutional Ethics Committee for Care and Use of Animals at Shiraz University of Medical Sciences.

***Isolation and primary culture of rat DRG***

**Figure 1:**
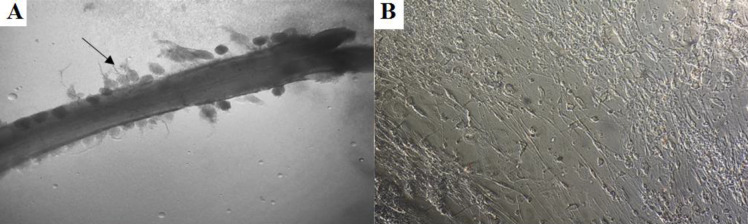
Dorsal root ganglion (DRG) isolated from rat embryos and DRG cells cultured *in vitro*

 The primary cultures of DRG neurons were isolated from Sprague-Dawley rats at the embryonic days of 14 and 15 under aseptic conditions (Figure 1A), as described previously (31,32). Briefly, DRG neurons were gently dissociated by enzymatic digestion (0.125%, Sigma-Aldrich, Saint Louis, MO) for 15 min at 37 °C and mechanical trituration. Resulting single cells were seeded on Petri dishes that were previously coated with poly-L-lysine hydrobromide (18 μg/mm^2^, 3 hr at room temperature). Cells were cultured in Neuro Basal-A medium supplemented with 2% B27 containing 2 mM GlutaMAX, 100 mg streptomycin, 100 units of penicillin, at 37 °C with 5% CO_2_ and 80% relative humidity. In order to remove non-neural cells, the day after incubation, cells were treated with antimitotic agent of 5-fluorodeoxyuridine (FUDR) and uridine at a final concentration of 20 mM for 72 hr.

***Immunocytochemistry***

In order to confirm the purity of the isolated DRG neurons, fluorescent anti-β III-tubulin (neuronal marker) monoclonal antibody and 4,6 diamidino-2-phenylindole (DAPI) dye, dual staining was performed. Firstly, cultured neurons on coverslips were washed twice with phosphate buffered saline (PBS) and then treated with ice-cold 3.7% formaldehyde in PBS for 10 min. Then, the cells were washed three times with 0.5% bovine serum albumin (BSA) and incubated in 5% normal goat serum (in 0.5% BSA solution) for 1 hr at room temperature. The cells were then washed twice with 0.5% BSA and incubated with mouse monoclonal antibody against _III-tubulin (Cell Signaling, 4466, 1:1000 in 0.5% BSA with 0.01% Triton X-100) for 1 hr at room temperature. The cells were then washed twice with 0.5% BSA and then incubated with secondary antibody conjugated with Alexa 488 (goat anti-mouse IgG, Cell Signaling, A-11029, 1:100 in 0.5% BSA with 0.01% Triton X-100) and 1 µg/ml DAPI (Sigma) for 30 min in the dark at room temperature. The cells were then washed three times with 0.5% BSA and photographed using a fluorescent microscope.

***Neurotoxicity assessment using MTT assay***

Cell viability was quantified using 3-(4,5-dimethyl-2-thiazolyl)-2,5- diphenyl-2H-tetrazolium bromide (MTT, Sigma) assay. Briefly, DRG neurons (1×10^4 ^cells/well) were plated in a 96-well plate and treated with various concentrations of DOX (0.4 to 100 µM), in three separate periods of 12, 24, and 48 hr. Cell viability was evaluated using MTT assay and the resulting data were analyzed by nonlinear regression (Prism 6 software) to calculate the IC_50 _value for DOX. To evaluate the possible protective effect of AM against DOX-induced toxicity, the cells were treated with various concentrations of AM (3.125 -100 nM) in the absence and presence of DOX (6.88 µM) for 24 hr and cell viability was evaluated using MTT assay. At the end of the incubation period, the media was replaced with MTT-containing media (0.5 mg/ml) and then incubated at 37 °C for 4 hr. An equal volume of dimethyl sulfoxide (DMSO) was added to solubilize formazan crystals and the absorbance of each well was measured at 570 nm. The experiment was carried out in triplicate in independent wells. The IC50 value for DOX was determined using Prism 6 software and was used in the subsequent experiments. For future examinations, DRG neurons were cultured at the density of 2×10^5^ cells/well in a 48-well plate and were classified as: control group (without any treatment), AM group (treated with 25 nM of AM), DOX group (treated with DOX at IC50 concentration 6.88 µM), and AM-DOX group (treated with 25 nM of AM and 6.88 µM of DOX), for 24 hr. In the AM-DOX group, AM was added to the wells 2 hr before the administration of DOX. 

***Apoptosis assay using annexin V and propidium iodide (PI)***

Flowcytometric apoptosis assay was performed using annexin V and propidium iodide (PI) double staining method (Apoptosis Detection Kit K-101-100, Bio Vision), according to the manufacturer’s instruction. Briefly, cells were plated in a 6-well plate at a density of 1 × 10^6^ cells/well and treated with DOX (6.88 µM) in the presence and absence of AM (25 nM) for 24 hr. The cells were then harvested and re-suspended in 500 μl binding buffer. A total of 5 μl PI and 5 μl annexin V were added and placed at room temperature for 5 min in the dark. Finally, the samples were analyzed using a flowcytometer (BD) at 488 nm.

***Measurement of ROS***

To measure intracellular ROS generation, we used the 2′, 7′-dichlorofluorescein diacetate (DCF-DA) dye. The non-fluorescent dye permeated cells easily and was hydrolyzed to fluorescent 2′, 7′- dichlorofluorescein upon interaction with intracellular ROS (33). Briefly, the cells were treated with DOX (6.88 µM) in the presence or absence of AM (25 nM) for 24 hr. At the end of the incubation period, the cells were treated with 10 μM of DCF-DA for 30 min at 37 °C under 5% CO_2_, were washed three times with PBS, and the fluorescence intensity was measured using an EnSpireR Multimode Plate Reader (Perkin-Elmer, Waltham, MA, USA), with an excitation wavelength of 488 nm and an emission wavelength of 525 nm. 

***Nitric-oxide assay***

We used the Griess-reduction colorimetric method to measure the NO level in the cell lysate of the studied groups, as described previously (34). Briefly, an equal volume of Griess reagent (0.1% naphthylethylenediamine and 1% sulfanilamide in 5 % H_3_PO_4_) was added to 200 μl of the samples and incubated at room temperature in the dark. Absorbance was determined at 540 nm using a microplate reader (Thermospectronic Genesys 5, Labequip, Ontario, Canada). Nitrite concentration was calculated using the standard curve of sodium nitrite (1−100 nmol/ml). 

***Measurement of malondialdehyde as a marker of lipid peroxidation***

Malondialdehyde (MDA) was measured using the thiobarbituric acid (TBA) method (35). All reagents for this assay were purchased from Merck (Darmstadt Germany). Briefly, the control and treated cells were lysed and placed into a 1.5 ml centrifuge tube with KCl (1.15%) solution. Then, 2000 μl of TBARS assay reagent was added to 500 μl of cell lysate and the solution was heated for 45 min in a boiling water bath. After cooling, 2 ml of n-butanol was added to each tube. The cell lysate was then centrifuged at 10000 × g for 10 min at 4 °C. The absorbance of the supernatant was measured using a microplate reader (Thermospectronic Genesys 5, Labequip, Ontario, Canada) at 532 nm.

***Determination of 8-iso-prostaglandin F2α (iPF2α) level***

The levels of 8-iso prostaglandin F2α (iPF2α), a biomarker of lipid peroxidation, were assayed using an ELISA Kit (Cayman, Item number. 51635) according to the manufacturer’s protocol. Briefly, the treated and control cells were lysed, and the esterified forms of iPF2α were released upon incubation with KOH 15% at 40 °C for 60 min. Thereafter, KH_2_PO_4_ 1.25 M was used to adjust the pH, and proteins were precipitated using a mixture of ethanol 30% containing butylhydroxytoluene 0.01%. The absorbance of the solution was determined at 405 nm. The protein concentration of the samples was determined using a BCA protein assay kit (Pierce, Rockland, IL). Data were normalized based on the protein concentration in each sample and expressed as a percentage of the control group. 

***Mitochondrial membrane potential (MMP) measurement***

Reduction in mitochondrial membrane potential (MMP) was measured using Rhodamine 123 (Rh 123). This cationic fluorescent dye accumulates in the mitochondria and loss of MMP results in the release of Rh 123 from the mitochondria and a decrease in intracellular fluorescence (36). Briefly, after 24 hr of incubation, the cells were washed with PBS and incubated with 1 μM of Rh 123 for 30 min at 37 °C. Afterward, the cells were washed with PBS and the fluorescence signal was quantified using an EnSpireR Multimode Plate Reader (Perkin-Elmer, Waltham, MA, USA), with an excitation wavelength of 488 nm and an emission wavelength of 525 nm. The data were normalized and expressed as a percentage of reduction in fluorescence intensity in comparison with the controls.

***Quantitative real-time polymerase chain reaction (RT-PCR)***

RT-PCR was used to evaluate the relative expression of AM, AM receptors components (CRLR and RAMP3), inflammatory mediators (IL-1β, TNF-α, iNOS), MMP-3, MMP-13, and SOX-9 in isolated neurons of DRGs. Briefly, total RNA was isolated using an RNA extraction kit (Qiagen, USA) according to the manufacturer’s protocol. cDNA was then synthesized**,** using 1,000 ng of total RNA in a first-strand cDNA synthesis reaction using RevertAid™ First Strand cDNA Synthesis kit (Thermo Fisher Scientific, Inc., Waltham, MA, USA). RT qPCR was performed using ABI Biosystems StepOne and the RealQ Plus 2x Master Mix Green (Ampliqon A/S, Odense, Denmark). In each reaction, 200 nM of each primer (Table 1) was added to target a specific sequence. cDNA fragments were amplified on a thermal cycler (Applied Biosystems, Thermo Fisher Scientific, Inc., USA) with thermal conditions set at 10 min at 94 °C followed by 40 cycles of 15 sec at 94 °C, 60 sec at 60 °C, and final extension of 7 min at 72 °C. Analysis of real-time PCR data was performed using the 2^-∆∆Ct^ method (Livak and Schmittgen, 2001) with target mRNA expression in each sample normalized against the endogenous control, the β-actin gene was used as the housekeeping gene.

***Statistical analysis***

Data were analyzed using the SPSS software package (version 21). All data are presented as means±SEM. One-way analysis of variance (ANOVA) followed by the Student-Newman-Keuls *post hoc* test was used to evaluate the presence of significant differences between groups. *P*<0.05 was considered to be statistically significant.

## Results

***DRG neurons were identified by anti-β-III-tubulin antibody and DAPI staining***

**Figure 2:**
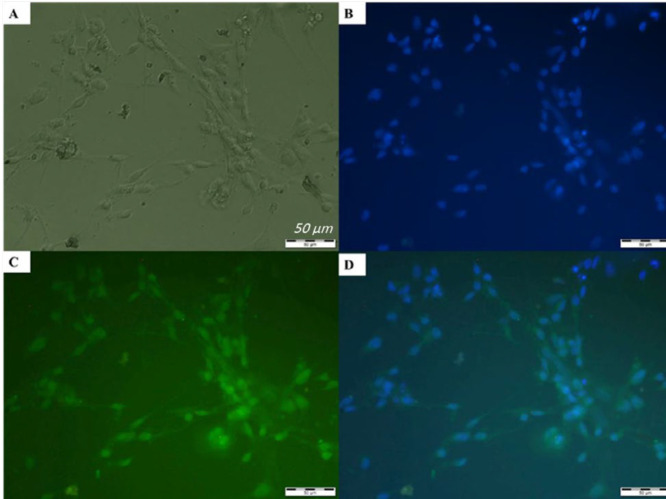
Immunofluorescence characterization of neurons derived from rat embryonic dorsal root ganglion (DRG) was done using fluorescent anti-β III-tubulin (neuronal marker, green) antibody. (A) Representative picture of DRG neurons taken by a phase-contrast microscope. The same cells were stained for DAPI (B) and anti-β III-tubulin (C). (D) Merged image of B and C (200 X). Scale bars: 50 µm

Monoclonal antibody against neuron-specific β III isotype of tubulin (anti-β III-tubulin) was used to determine the purity of the primary neuron culture of rat DRGs. The stained DRG cells by DAPI (blue fluorescence) and anti-β III-tubulin (green fluorescence) are presented in Figures 2B and 2C, respectively. Merging of Figures 2B and 2C is shown in Figure 2D. The number of β-tubulin III-positive cells was counted in 3 fields from 3 different coverslips. Based on the quantification, the population of β III-tubulin expressing cells was 92±7 %, indicating an acceptable purity of neuronal cells in the cultured cells. 

***AM reduced DOX-induced cell death***

**Figure 3:**
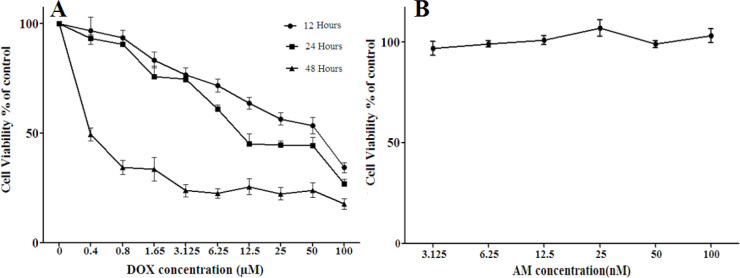
The effects of doxorubicin (DOX) and adrenomedullin (AM) on cell viability of DRG neurons. A: Dorsal root ganglion (DRG) neurons were treated with various concentrations of DOX (0.4-100 µM) for 12, 24, and 48 hr, and cell viability was measured using MTT assay. B: The effects of AM treatment (3.125-100 nM for 24 hr) on cell viability of DRG neurons. The presented data are mean±EM of at least six independent experiments. One-way analysis of variance (ANOVA) followed by the Student-Newman-Keuls *post hoc* test was used to evaluate the presence of significant differences between groups. In all used concentrations (0.4-100 µM) and incubation times, DOX reduced cell viability in a concentration-dependent manner. AM had no significant effect on cell viability in concentrations of up to 100 Nm

To evaluate the possible protective effect of AM against DOX-induced neurotoxicity, cells were treated with various concentrations of DOX (0-100 µM) in the presence and absence of AM. MTT results indicated that DOX reduced cell viability of DRG neurons in a dose and time-dependent manner (Figure 3A). IC50 values of DOX were calculated as 17.864±2.37, 6.888 ± 0.6, and 0.185 ± 0.02 µM for 12, 24, and 48 hr of treatment, respectively. AM alone had no significant effect on the viability of cultured DRG neurons at concentrations up to 100 nM (Figure 3B). 

Pre-treatment with various concentrations of AM significantly ameliorated DOX (6.888 µM for 24 hr) -induced cell death, and increased cell viability in a concentration-dependent manner. The ameliorating effects of AM against DOX-induced neurotoxicity revealed significant differences at concentrations of 25, 50, and 100 nM (*P*-value< 0.0001). Based on these findings, the concentrations of 6.888 µM of DOX and 25 nM of AM were selected to be used in further experiments. 

***The effects of AM on DOX-induced apoptosis of DRG neurons***

Annexin V /PI staining and flowcytometry analysis were used to quantify the effects of AM on DOX-induced cell death of DRG neurons. The data shows that treatment of DRG neurons with DOX (6.88 µM, for 24 hr) increased the percentage of late apoptotic as well as necrotic cells. As presented in Figure 5E, the percentage of viable cells (Q1), significantly decreased in the DOX group, compared with the untreated cells; however, pre-treatment with AM reversed DOX-related viability, *P*-value<0.05. Furthermore, pre-treatment with AM (25 nM) significantly (*P*-value<0.0001) reduced DOX-induced cell death and apoptosis (Figure 5). 

***DOX treatment reduced the expression of AM and its receptor components***

The effect of AM and/or DOX treatments on the expression of AM and its receptor components (CLR and RAMP3) are shown in Figure 6. As expected, AM treatment reduced its own expression at mRNA level compared with the control group (*P*-value<0.0001), which was probably through a negative feedback mechanism. A significant decrease in the levels of AM, CLR, and RAMP3 mRNAs was observed in the DOX-treated group compared with the control group. AM pre-treatment reversed the effect of DOX on AM, CLR, and RAMP mRNAs levels (Figure 6). 

***AM ameliorates DOX-induced oxidative stress***

The oxidative stress marker levels were measured to explore the possible antioxidant effects of AM against DOX-induced neurotoxicity (Figure 7). As seen in Figure 7A, the ROS level significantly increased following treatment with DOX compared with the control group (*P*-value<0.0001). Pretreatment with 25 nM of AM significantly decreased ROS level compared with cells that were treated with DOX alone (*P*-value<0.0001). Furthermore, DOX significantly increased the NO level in comparison with untreated cells (*P*-value=0.0520). AM pretreatment led to a significant decrease in DOX-induced NO production, in comparison with the DOX group (*P*-value=0.03). Finally, a significant increase in MDA and iPF2α levels was observed in the DOX-treated group compared with the control group (*P*-value=0.0063). Pre-treatment with AM decreased the elevated levels of MDA and iPF2α compared with the DOX group (*P*-value=0.0063 and *P*-value=0.006, respectively). 

***Effect of AM on DOX-induced MMP dissipation***

Decreased MMP is associated with mitochondrial dysfunction and consequent cell death. The MMP level significantly declined in the DOX-treated group compared with untreated cells (*P*-value=0.004). Co-treatment with AM (25 nM) significantly reduced the effect of DOX on loss of MMP (*P*-value=0.004). AM treatment alone did not affect the MMP level. 

***AM reversed the effects of DOX on the expression of IL 1β, TNF-α, iNOS, and Sox9***

As shown in Figure 9A, DOX treatment increased IL-1β (*P*=0.0036), TNF-α (*P*-value<0.001), and iNOS (*P*-value=0.0029) mRNA levels compared with those of the control group. AM pre-treatment significantly reversed the effects of DOX on the expression of IL 1β, TNF-α, and iNOS. As presented in Figure 9D, DOX decreased the level of Sox9 mRNA (*P*-value=0.004) while pre-treatment with AM for 2 hr, reversed the effects of DOX on Sox9 mRNA (*P*-value=0.004).

***Effect of AM on MMP3 and MMP13 gene expression***

A significant increase in the levels of MMP3 and MMP13 was observed in the DOX treated groups compared with the control group (*P*-value=0.004 and *P*-value=0.0145, respectively). AM pre-treatment decreased DOX-induced MMP3 mRNA levels (*P*-value=0.0004) while it had no significant effect on MMP13 mRNA levels (Figures 10 A and B). 

DRGs were treated with DOX (6.88 µM) in the absence and presence of AM (25 nM) for 2 hr. The levels of MMP3 and MMP13 were determined using real-time PCR. Relative expression of each mRNA compared with β-actin mRNA was calculated in control and treated groups. The data are reported as a percentage of the control group. Data were obtained from six independent experiments performed in triplicate and expressed as mean ± SEM. One-way analysis of variance (ANOVA) followed by the Student-Newman-Keuls *post hoc* test was used to analyze the data. * and # reveal significant difference (*P*-value<0.05) compared with control and DOX groups, respectively.

## Discussion

In the present study, purified embryonic DRG neurons were used as an *in vitro* model to evaluate the protective effects of AM against DOX-induced neurotoxicity. Our data revealed ameliorating effects of AM against DOX-induced toxicities, including cell death, apoptosis, oxidative stress, and expression of inflammatory mediators in the DRG neurons. These findings suggest that AM can be considered a pharmacological agent to ameliorate the neurotoxic effects of DOX in cancer patients.

In a recent study by Yoshizawa *et al*. (30), a significant decrease in the expression of CLR was observed, following exposure of cardiac tissue to DOX. In line with this finding, our data showed a significant decrease in AM, CLR, and RAMP3 mRNA levels in DOX treated DRG cells, suggesting a decrease in AM signaling pathway in the pathogenesis of DOX-induced neurotoxicity. Interestingly, administration of AM increased its own gene expression, as well as its receptor component (CLR/ RAMP3) expression in the DOX treated cells, indicating the stimulatory effect of AM on its own signaling pathway through transcriptional activation of CLR/RAMP genes, a phenomenon that was previously described in human microvascular endothelial cells (37).

Our data from MTT assay clearly revealed that DOX administration is associated with cell death in the DRG neurons in a time and dose-dependent manner. These findings are in agreement with that of Manchon *et al*. study on rat embryonic cortical neurons (38), indicating the toxicity of DOX on primary sensory neurons. The results of the apoptosis assay revealed that exposure to DOX increased both apoptosis and necrosis of DRG cells, which is in line with the results of previous studies (39, 40). It was reported that exogenous AM can protect neurons against cerebral ischemia-induced apoptosis (41). In consistence with this report, our findings indicated that pretreatment with AM was able to reverse DOX-induced cell death, predominantly through reducing DRG neuron necrosis. 

Overproduction of ROS and RNS and the consequent oxidative damage as well as inflammatory reactions are proposed as the main mechanisms responsible for DOX-induced toxicity (7). In accordance with the findings of previous studies (42, 43), our results revealed alteration in the levels of various aspects of oxidative stress, including elevated levels of ROS, NO, MDA, and iPF2α in association with MMP dissipation in the DOX-treated DRG neurons. Antioxidant properties of AM were shown in numerous conditions (20, 44). In the current study, we observed that AM pretreatment was able to suppress the oxidizing effects of DOX in DRG neurons, evidently through lowering the levels of ROS, NO, MDA, and iPF2α. Although the underlying mechanism was not investigated in our study, modulation in the enzymatic activities of antioxidant enzymes and increase in the supply of glutathione (GSH) are the proposed mechanisms in this context (45). In agreement with previous studies, our data revealed a significant MMP dissipation following DOX exposure. In cardiac muscle cells, it was shown that AM treatment could prevent DOX-induced damage to the inner mitochondrial membrane (30). Therefore, protecting neuronal mitochondria against the DOX effect is another mechanism that might be involved in antioxidant effects of AM.

It is evident that DOX-induced toxicities are closely linked to increased expression of inflammatory mediators, such as TNF-α, IL-1β, IL-6, and iNOS (46). In line with these studies, our data revealed an increase in the expressions of TNF-α, IL-1β, and iNOS, following the exposure of DRG cells to DOX, suggesting a possible role for these inflammatory mediators in DOX-induced toxicities in the DRG neurons. In agreement with several investigations in various inflammatory models (21, 46), our data showed that pretreatment with AM reduced DOX-induced expressions of TNF-α, IL-1β, and iNOS in the cultured DRG, suggesting the anti-inflammatory function of AM against DOX-induced toxicity.

SOX9 is a multifunctional transcription factor that plays a crucial role in various physiological processes, including CNS development (47) and maintenance of neural stem cells (48). It has been shown that DNA damage induced by genotoxic agents, such as DOX, cisplatin, and UV-irradiation leads to degradation of SOX9 by proteasomes (16) while its upregulation leads to the prevention of cellular senescence and apoptosis, suggesting the role of this protein in cell survival (49). Our results showed that treatment of embryonic DRG neurons by DOX caused a significant decrease in Sox9 mRNA expression level. We also observed that AM was able to reverse the decreased expression of SOX9 in DOX-treated DRG neurons, which could be regarded as a possible mechanism of AM neuroprotection property. It was shown that SOX9 expression was increased transcriptionally by binding of Sp-1 and CREB to their binding sites in the promoter of SOX9 gene (50) while SOX9 expression was down-regulated by IL-1β and TNF- α (51) through inhibiting the association of Sp1 to its binding site in SOX9 promoter. BMP-2 might also be involved in the increase of SOX9 transcription following AM treatment since it was previously reported that AM increased BMP-2 in the DRG through the induction of histone hyperacetylation at the SOX9 locus (52).

The association of MMPs including MMP-3 and -13 with chemotherapy-induced cell toxicities, such as mucositis (53), glomerulosclerosis (54), and neurotoxicity (55) was implicated. Our findings revealed a significant increase in mRNA levels of MMP-3 and MMP-13 following treatment of DRG neurons with DOX. Increased MMP-3 expression also revealed that following rat DRG neuron exposure to paclitaxel (56), suggested the possible role of MMP-3 in the chemotherapeutic drug-induced neurotoxicity. Our data revealed a significant reduction in MMP-3 mRNA level in the DOX-treated DRG cells that were pretreated with AM compared with cells that were treated with DOX alone. Previous studies reported the critical role of ROS (16) and inflammatory mediators (56) in the activation of MMPs, suggesting the possible role of antioxidant and anti-inflammatory properties of AM. However, further studies are warranted to shed more light on the exact underlying mechanisms.

Despite beneficial effects of antioxidants against chemotherapy-induced toxicity, the investigator’s view about the use of antioxidants during chemotherapy is controversial. Some believe that antioxidants can reduce chemotherapy effectiveness; therefore, they prohibit patients from taking antioxidants during chemotherapy. On the other hand, others recommend the use of antioxidants during chemotherapy, since they claim that the protective effect of antioxidants is cell-specific and is restricted to normal cells (57). In the present study, AM ameliorated DOX-induced toxicity in normal DRG neurons, but whether in tumor cells AM functions similarly by protecting the cells against DOX toxicity or the opposite by enhancing the effectiveness of DOX is still debatable and further investigations are warranted.

A) Spinal cord isolated from rat embryos at embryonic days of 14 and 15 (arrow shows DRGs within the spinal canal. B) A representative view of embryonic rat DRG cells before treatment with anti-mitotic agent

**Table 1:** Primer sequences for reverse transcription-polymerase chain reaction of adrenomeullin and inflammatory mediators

**No**	**Gene **	**Accession number**	**Primer sequence (5'3')**	**Size(bp) **
	AM	NM_012715.1	Forward:GAACAACTCCAGCCTTTACC Reverse:GAGCGAACCCAATAACATCAG	62
	CRLR	NM_012717.1	Forward:CACACCAAGCAGAATCCAATC Reverse:GTCATACACCTCCTCAGCAA	59.3
	RAMP3	NM_020100	Forward:CTGACCTCTGCTACGCTTG Reverse:TGACTCCTAACAACTCCATTCC	62.2
	IL1β	BC091141.1	Forward:GGAGAGACAAGCAACGACAA Reverse:TTGTTTGGGATCCACACTCTC	123
	TNF-α	AF269159.1	Forward:CCCACGTCGTAGCAAACCAC Reverse:TAGGGCAAGGGCTCTTGATG	264
	iNOS	AABR07030077.1	Forward:GGATGTGGCTACCACTTTGA Reverse:CATGATAACGTTTCTGGCTCTTG	107
	SOX-9	AB073720.1	Forward:AGTCGGTGAAGAATGGGCAA Reverse:ACCCTGAGATTGCCCGGAG	161
	MMP-3	X02601.1	Forward:ATGATGAACGATGGACAGATGA Reverse:CATTGGCTGAGTGAAAGAGACC	99
	MMP-13	M60616.1	Forward:CAAGCAGCTCCAAAGGCTAC Reverse:TGGCTTTTGCCAGTGTAGGT	130

**Figure 4:**
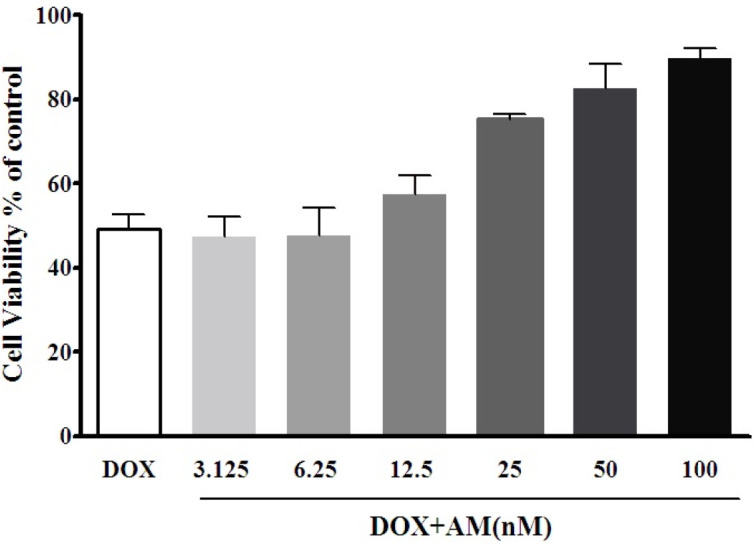
Protective effect of adrenomedullin (AM) against doxorubicin (DOX)-induced cell death on DRG neurons. DRG neurons were treated with DOX (6.88 µM) in the absence and presence of various AM concentrations (3.125-100 nM) for 24 hr, and cell viability was evaluated using MTT assay. The represented data are mean±SEM of at least six independent experiments. One-way analysis of variance (ANOVA) followed by the Student-Newman-Keuls *post hoc* test was used to analyze the data. AM (12.5 up to 100 nm) increased cell viability of DOX-treated cells dose-dependently (*P*<0.05)

**Figure 5:**
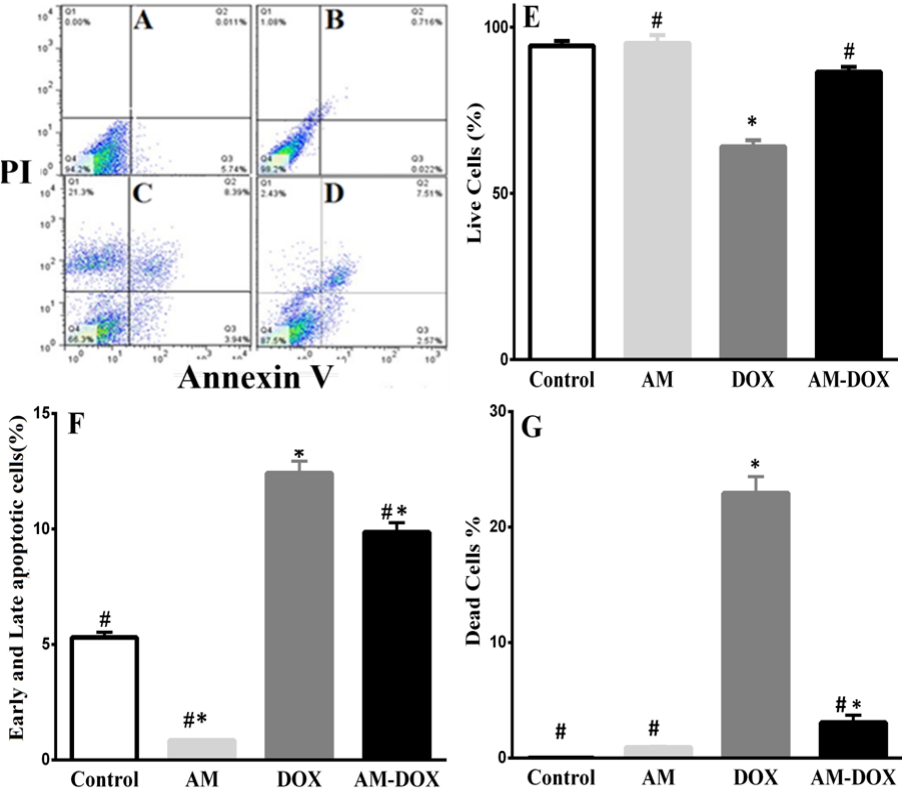
The effect of adrenomedullin (AM) on doxorubicin (DOX)-induced apoptosis of Dorsal root ganglion (DRG) neurons. The cells were treated with DOX (6.88nM) in the absence or presence of AM (25 nM) for 24 hr, and apoptosis was measured using annexin V /PI and flow cytometry method. A, B, C, and D represent the flow cytometric scatter diagram for control, AM, DOX, and AM-DOX treated groups, respectively. E, F, and G bar charts represent the percentages of live, total apoptotic (early + late) cells, and dead cells in various groups, respectively. The presented data are mean±SEM of at least three independent experiments. One-way analysis of variance (ANOVA) followed by the Student-Newman-Keuls *post hoc* test was used to analyze the data. * and # show significant difference (*P*-value<0.05) compared with control and DOX groups, respectively

**Figure 6:**
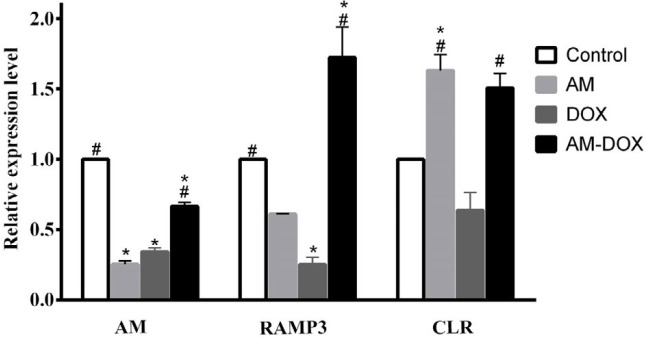
Effect of adrenomedullin (AM) on mRNA levels of AM, receptor activity modifying protein 3 (RAMP3), and calcitonin receptor-like receptor (CLR) in DOX-treated dorsal root ganglion (DRG) neurons. DRG neurons were treated with doxorubicin (DOX) (6.88 µM) in the absence or presence of AM (25 nM) for 24 hr. AM, RAMP3, and CLR levels were determined using real-time PCR. Relative expression of each mRNA compared with β-actin mRNA was calculated in control and treated groups. Data were obtained from six independent experiments performed in triplicate and expressed as mean±SEM. One-way analysis of variance (ANOVA) followed by the Student-Newman-Keuls *post hoc* test was used to analyze the data. * and # reveal significant difference (*P*-value<0.05) compared with control and DOX groups, respectively

**Figure 7:**
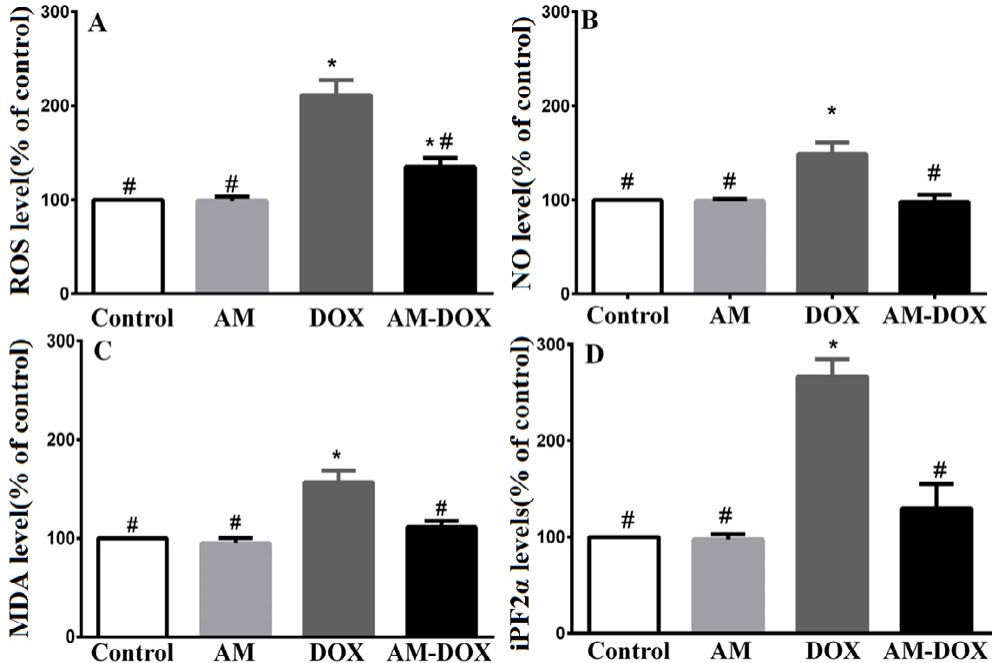
Effect of adrenomedullin (AM) on the levels of reactive oxygen species (ROS), NO, malonedialdehyde (MDA), and iPF2α in doxorubicin (DOX)-treated dorsal root ganglion (DRG) neurons. DRG neurons were treated with DOX (6.88 µM) with and without AM (25 nM) pretreatment. The levels of ROS, NO, MDA, and iPF2α were determined as described in the methods section. The data are reported as a percentage of the control group. Data were obtained from six independent experiments performed in triplicate and expressed as mean±SEM. One-way analysis of variance (ANOVA) followed by the Student-Newman-Keuls *post hoc* test was used to analyze the data. * and # reveal significant difference (*P*-value<0.05) compared with control and DOX groups, respectively

**Figure 8:**
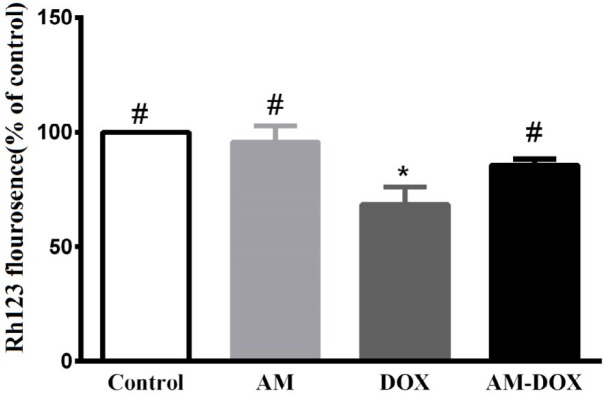
Effects of AM on DOX-induced MMP dissipation in dorsal root ganglion (DRG) neurons

DRG neurons were treated with DOX (6.88 µM) in the absence and presence of AM (25 nM) for 24 hr. MMP was measured using Rhodamine 123 method. Each value represents the mean±SEM determined in three independent experiments. One-way analysis of variance (ANOVA) followed by the Student-Newman-Keuls *post hoc* test was used to analyze the data. * and # reveal significant difference (*P*-value<0.05) compared with control and DOX groups, respectively

**Figure 9:**
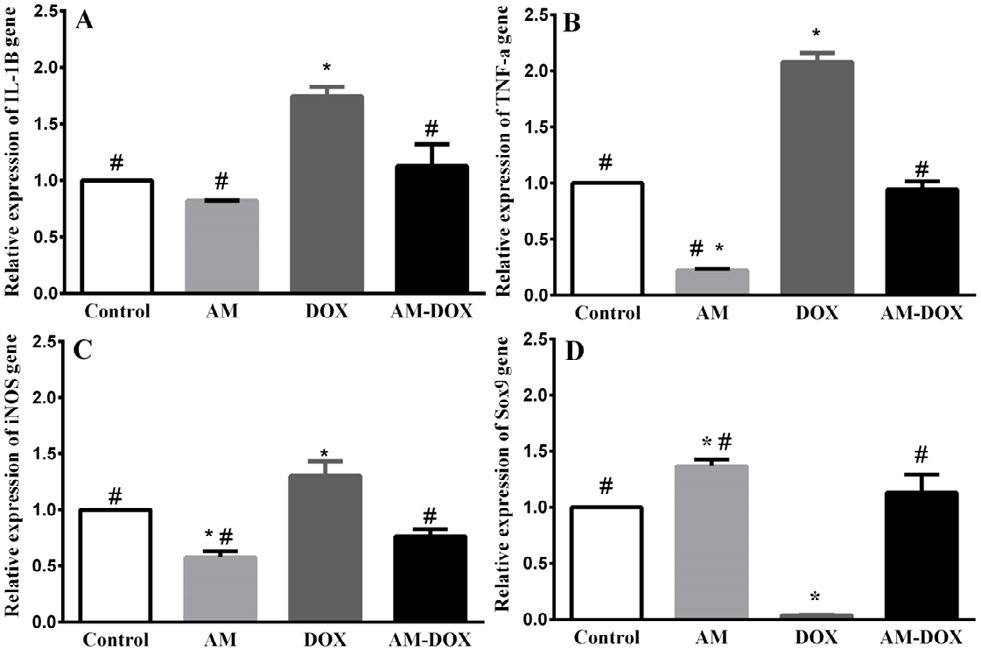
Effect of adrenomedullin (AM) on doxorubicin (DOX)-induced gene expressions of IL-1β, TNF-α, iNOS, and SOX9. DRGs were treated with DOX (6.88 µM) in the absence and presence of AM (25 nM) for 2 hr. The levels of IL-1β, TNF-α, iNOS, and SOX9 were determined using real-time PCR. Relative expression of each mRNA compared with β-actin mRNA was calculated in the control and treated groups. The data are reported as a percentage of the control group. Data were obtained from six independent experiments performed in triplicate and expressed as mean±SEM. One-way analysis of variance (ANOVA) followed by the Student-Newman-Keuls *post hoc* test was used to analyze the data. * and # reveal significant difference (*P*-value<0.05) compared with control and DOX groups, respectively

**Figure 10:**
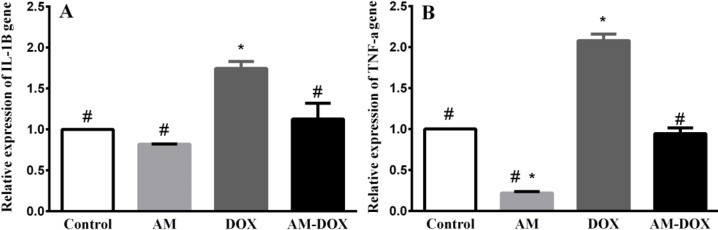
Effect of doxorubicin (DOX) and adrenomedullin (AM) on matrix metalloproteinase 3 (MMP3) and MMP13 mRNA levels

DRGs were treated with DOX (6.88 µM) in the absence and presence of AM (25 nM) for 2 hr. The levels of MMP3 and MMP13 were determined using real-time PCR. Relative expression of each mRNA compared with β-actin mRNA was calculated in control and treated groups. The data are reported as a percentage of the control group. Data were obtained from six independent experiments performed in triplicate and expressed as mean±SEM. One-way analysis of variance (ANOVA) followed by the Student-Newman-Keuls *post hoc *test was used to analyze the data. * and # reveal significant difference (*P*-value<0.05) compared with control and DOX groups, respectively

## Conclusion

Our study showed the protective effect of AM against DOX-induced toxicity in embryonic DRG neurons of rats, a phenomenon that might be related to antioxidant and anti-inflammatory properties of AM. All in all, this study suggests that AM can be considered a novel ameliorating drug against chemotherapeutic agent-induced toxicity. 
